# Testing of Visual Field with Virtual Reality Goggles in Manual and Visual Grasp Modes

**DOI:** 10.1155/2014/206082

**Published:** 2014-06-23

**Authors:** Dariusz Wroblewski, Brian A. Francis, Alfredo Sadun, Ghazal Vakili, Vikas Chopra

**Affiliations:** ^1^BioFormatix, Inc., P.O. Box 721450, San Diego, CA 92172, USA; ^2^Department of Ophthalmology, Keck School of Medicine, University of Southern California, Los Angeles, CA 90033, USA; ^3^Doheny Eye Institute, Department of Ophthalmology, University of California Los Angeles (UCLA), Los Angeles, CA 91105, USA

## Abstract

Automated perimetry is used for the assessment of visual function in a variety of ophthalmic and neurologic diseases. We report development and clinical testing of a compact, head-mounted, and eye-tracking perimeter (VirtualEye) that provides a more comfortable test environment than the standard instrumentation. VirtualEye performs the equivalent of a full threshold 24-2 visual field in two modes: (1) manual, with patient response registered with a mouse click, and (2) visual grasp, where the eye tracker senses change in gaze direction as evidence of target acquisition. 59 patients successfully completed the test in manual mode and 40 in visual grasp mode, with 59 undergoing the standard Humphrey field analyzer (HFA) testing. Large visual field defects were reliably detected by VirtualEye. Point-by-point comparison between the results obtained with the different modalities indicates: (1) minimal systematic differences between measurements taken in visual grasp and manual modes, (2) the average standard deviation of the difference distributions of about 5 dB, and (3) a systematic shift (of 4–6 dB) to lower sensitivities for VirtualEye device, observed mostly in high dB range. The usability survey suggested patients' acceptance of the head-mounted device. The study appears to validate the concepts of a head-mounted perimeter and the visual grasp mode.

## 1. Introduction

Automated perimetry is a noninvasive technique for evaluating pathology or dysfunction in the visual pathways. It has proven to be a fundamental method for the detection, characterization, and monitoring of progression for a variety of ophthalmic and neurologic diseases.

In the basic perimetry measurement, the patient is presented with a series of light stimuli of varying intensities in different visual field locations, and the threshold sensitivity of the retina is assessed based on the patient's ability to consciously detect and respond to these stimuli. Utilizing this test, vision loss can be detected and often localized to a specific anatomic location. Moreover, the pathology can be followed in a longitudinal fashion to detect progression of disease.

Various implementations of perimetry have been used for more than 100 years to assess vision abnormalities [1]. Kinetic perimetry maps the visual field by using moving targets of varying size and brightness. Static perimetry, which is employed in modern standard automated perimetry (SAP) devices, uses light targets of varied luminance at preset locations typically within the central 30 degrees of the visual field (VF) [2]. This enables accurate measurement of the differential light sensitivity of the target against a constant illuminated background. The SAP measurements are sensitive to localized and shallow depressions (relative scotomas) in the visual field, such as those seen in glaucoma. Standardization of tests enables comparison and statistical analysis. During the last few decades, perimetry devices have been improved considerably. The recent technological advances in the visual field testing focus mainly on employing novel visual stimuli that are deemed to be more effective in detecting glaucoma [3, 4]. These newer methods include frequency-doubling technology (FDT) [5] and color-on-color or short-wavelength automated perimetry (SWAP) [6].

The SAP test may be stressful for a patient as it requires maintenance of constant fixation for several minutes and conscious decision making in identification of near-threshold stimuli. The inherently subjective nature of the test complicates the test interpretation task [7]. For example, a patient's psychophysical status will influence his/her alertness level and reaction to the visual stimuli. This variability may occur from examination to examination (long-term fluctuation or variance) or even within the same test (short-term fluctuation) [8, 9]. The test fluctuations are often higher in glaucoma patients, making it more difficult to assess stability or progression of VF defects.

Head-mounted perimetry devices have been considered previously, especially for immobilized patients and out-of-clinic use [10, 11]. Potential benefits include an increase in the patient's comfort and potential decrease in the test-induced fatigue and may be useful in testing patients with cervical or spinal disease that makes positioning in a standard perimeter difficult. The visual grasp mode studied here is analogous to the previously proposed eye motion perimetry [12]. It is more reflexive, as it does not require manual input or the cortical processing required for the standard mode.

## 2. Materials and Methods

### 2.1. Head-Mounted Virtual Reality Display with Eye Tracker

**Figure 1:**
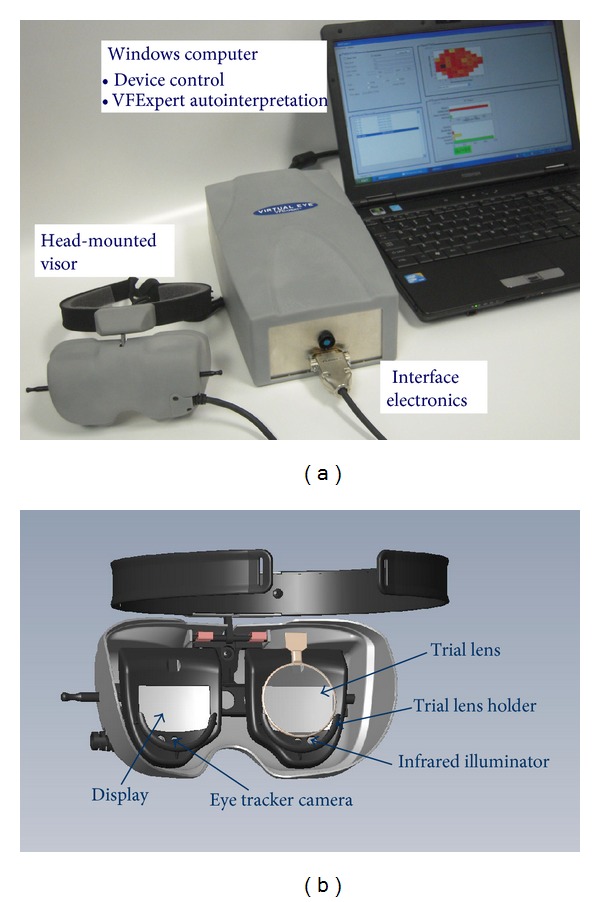
(a) Components of portable VirtualEye perimeter. (b) Back view of VirtualEye head-mounted visor. The device is secured on patient's head with adjustable headband (top of the picture).

The VirtualEye head-mounted apparatus for visual field testing is shown in Figure 1. The device integrates microdisplays and a binocular eye tracker enclosed in a head-mounted visor and an interactive computer program for data collection and interpretation. The total weight of the prototype head-mounted device (display, eye tracker, and visor) is approximately 200 grams, with cable adding about 60 grams.

The VirtualEye perimeter was designed to emulate the performance of standard instruments such as the Humphrey field analyzer (HFA II) from Carl Zeiss Meditec (Dublin, CA, USA), or Easyfield from Oculus (Wetzlar, Germany). It provides a full threshold test using a 24-2 grid of test points. The sensitivity range is from 0 to 40 dB (decibels). Two test modes are available: manual (push button) response and hands-free visual grasp response.

The essential hardware components are as follows (Figure 1(b)).

1. OLED (organic light-emitting diode) microdisplays with compact viewing optic: we employed eMagin (Bellevue, WA) SVGA XL monochrome (green) OLED displays. OLED displays are active (light-emitting) as opposed to the most often employed passive (back-lit) liquid crystal displays (LCDs) and are thinner and lighter than LCDs. Their high luminance and angle-independent (Lambertian) light output make them suitable for the present application [13].
2. Binocular ViewPoint Eye Tracker system from Arrington Research (Scottsdale, Arizona): the system uses infrared video tracking with a temporal resolution of 60 Hz. The nominal angular resolution (the smallest change in eye position that can be measured) is about 0.15°. Eye tracker enables real-time monitoring of gaze direction (fixation). In the visual grasp mode, the tracker is used to capture responses to the light stimuli, thus providing a hands-free mode of operation that relies on the visual grasp reflex.

For patients requiring far-field correction, standard trial lenses are accommodated by lens holders—Figure 1(b). As shown in the figure, the tracker hardware, consisting of two infrared diode illuminators and two infrared miniature cameras, is mounted in a fixed position with regard to the OLED displays. The visor is adjusted with respect to subject's eyes using double-pivot mechanism placed between the headband and the visor (similar to eMagin Z800 3D visor). Intraocular distance may be adjusted with push-pull slide mechanism that permits independent movement of each display assembly in the horizontal direction. These adjustments are sufficient to accommodate a variety of facial structures and to assure proper positioning of the visor with respect to patient's eyes. The adjustments may be performed either by the patient or by the technician (who can see the images of patient's eyes on the computer monitor).

### 2.2. User Interface

**Figure 2:**
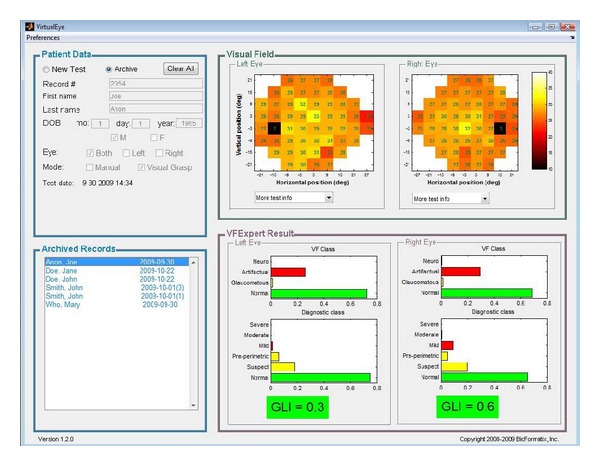
Graphical user interface (Windows) for VirtualEye perimeter.

The device is operated through a portable Windows computer (laptop or desktop). A simple, single-screen graphical user interface (Figure 2) provides access to the essential functionalities (four panels, clockwise from upper left): (1) test setup, input and display of patient's personal information, (2) color-coded display of visual field measurements, (3) VFExpert automatic interpretation of visual field measurements [14], and (4) access to archived data.

### 2.3. Instrument Capabilities

VirtualEye performs the equivalent of full threshold 24-2 test in manual and visual grasp modes. Briefly, the full threshold 4/2 strategy is implemented as follows. Starting with expected sensitivity for a given test point, the following stimulus is made 4 dB darker if there was positive response or 4 dB brighter if there was no response. The 4 dB steps continue until there is an opposite response (i.e., negative or positive, resp.) at which point 2 dB steps are applied in the opposite direction (i.e., the stimuli are made brighter or darker). The brightness of the last detected stimulus determines the threshold sensitivity. The expected sensitivity is initially set to 30 dB. As sensitivity values are acquired for consecutive test points, the expected sensitivity is estimated as weighted average of already measured sensitivities (weighted by inverse distance from the current test point). The use of this rough estimate results in shortening of the test time.

Either one or both eyes may be tested during a single session. When both eyes are tested, the light stimuli are produced alternately on the right or left display and the patient is usually not aware which eye is tested at a given moment. Both displays are active for single eye testing but stimuli are produced on one screen only (eye patch is not required).

The field of view for currently used OLED displays and imaging optic is 40 degrees diagonally (total field of view of 32 degrees and 24 degrees in the horizontal and vertical directions, resp.). In order to increase the angular coverage, we use the dynamic fixation approach, in which the fixation point is moved during the test. Thus, a fixation point located at the left edge of the display produces available horizontal field of view of 32 degrees, albeit only on the right-hand side of the fixation point.

Only the standard 24-2 test, with size III stimulus and stimulus presentation time of 0.2 sec, is available in the current VirtualEye prototype. However, the device is designed for maximum flexibility in implementation of different test scenarios. All crucial test parameters, including test pattern, stimulus size, and presentation time, are easily modified by editing relevant (internal) software parameters. Thus, any test pattern (such as 10-2, high resolution, or nonconventional patterns) can be easily implemented if needed, as long as it remains within the available spatial coverage. Implementation of different test strategies (such as SITA) would require inclusion of dedicated software modules.

*Manual mode* is akin to the standard perimeter test scenario. The patient is instructed to maintain steady fixation and responds to a visible stimulus by a mouse click. Lack of mouse click indicates no response or a stimulus that is not visible to the patient. The fixation point is presented consecutively at nine predetermined positions (center, middle of 4 edges, and 4 corners).

*Visual grasp* mode takes advantage of the natural tendency to look at a new, moving, or transient visual stimulus. It is thought that the M-cell system inputs to a reflex that, unsuppressed, drives an eye movement to acquire the novel target to the fovea. This mode does not require a manual input from the patient and does not require the cortical processing and decision making required for manual mode.

The eye tracker is used to detect the changes in gaze direction associated with presentation of the stimuli. In the visual grasp mode, the patient is instructed to first look in the direction of the fixation target and then, if the stimulus is visible, in the direction of the stimulus. The positive detection is determined from analysis of tracker data: if a patient's gaze change is consistent with the position of the stimulus with respect to the current fixation point, positive detection is deduced. When the stimulus is detected, the fixation point moves to the position of the detected stimulus (i.e., the stimulus location becomes the new fixation point). If the stimulus is not detected, the fixation point remains in the same spot and another stimulus is presented.

The perimeter software provides detection of false positive and false negative responses in both modes. Test data such as test duration, number of presented stimuli, number of false positive and false negative responses, test date, and others are automatically stored. These data may be recalled for viewing, together with sensitivity measurement data, through* Archived Records* panel (see Figure 2).

The test result is displayed in the* Visual Field* panel of the user interface. The panel is updated during the test, as testing of individual locations is completed, allowing the operator to follow the test progress. Complete result is displayed when the test is completed. The panel shows values of retinal sensitivity (in decibel, dB), color-coded according to the scale shown on the right-hand side of the panel. The actual dB values are also shown at the relevant locations. If both eyes were tested during a single test, the result for both eyes would be displayed. Otherwise, only the tested eye is shown.

The test results are sent automatically to VFExpert program and the analysis results are displayed in the* VFExpert Result* panel of the user interface. As previously described, VFExpert provides a standardized assessment of glaucoma using only the results of visual field test [14]. The program is based on a database of over 2000 patients, with classifications provided by team of glaucoma experts. The visual field data are automatically classified into (1) visual field class, and (2) glaucoma diagnosis class. The Visual Field classes include normal, glaucomatous, artifactual, and neurologic. The probability of a membership in each class is indicated graphically by the length of corresponding bars on the classification result figure. Only those fields that are classified as normal or glaucomatous can be meaningfully classified in terms of glaucoma diagnostic class. The Glaucoma Diagnosis classes include normal, suspect, preperimetric, mild glaucoma, moderate glaucoma, and severe glaucoma. Glaucoma likelihood index (GLI) provides a single-number summary of Visual Field examination. The range of GLI is from 0 (normal) to 5 (severe glaucoma).

### 2.4. Clinical Testing

The VirtualEye instrument was tested at the Doheny Eye Institute, Department of Ophthalmology, Keck School of Medicine, University of Southern California (Los Angeles, CA). Research was approved by the USC Institutional Review Board and adhered to the Declaration of Helsinki regarding research on human subjects and the Health Insurance Portability and Accountability Act (HIPAA). All patients undergoing treatment at the Doheny Eye Institute were considered for participation, regardless of age, sex, ethnicity, or health status. Persons younger than 18 years were not included because of issues regarding informed consent.

Test subjects were recruited from the glaucoma and neuroophthalmology clinics and were assessed in two roles: (1) clinical evaluation of the VirtualEye device and its comparison with the “gold standard” and (2) focus group to aid in usability testing (the patients were also asked to comment on possible improvements in the system design and operation).

The participants were tested at least once with the standard HFA II instrument (Carl Zeiss, Meditec) and twice with the VirtualEye device. SITA or SITA Fast full threshold strategy was used with HFA II. The VirtualEye testing included manual and visual grasp modes and only one eye was tested in each patient. The tested eye was generally selected as the one with larger visual field defects (as determined by previous HFA II exams). All recruited subjects had previous experience with HFA II testing and thus were tested first with HFA II and then with VirtualEye. Also, in majority of cases the first VirtualEye test was performed in manual mode and the second in visual grasp mode. In order to familiarize the subjects with the visual grasp procedure, a short “trial” run of visual grasp test was performed before the actual test.

## 3. Results

### 3.1. Display Performance and Luminance Calibration

**Figure 3:**
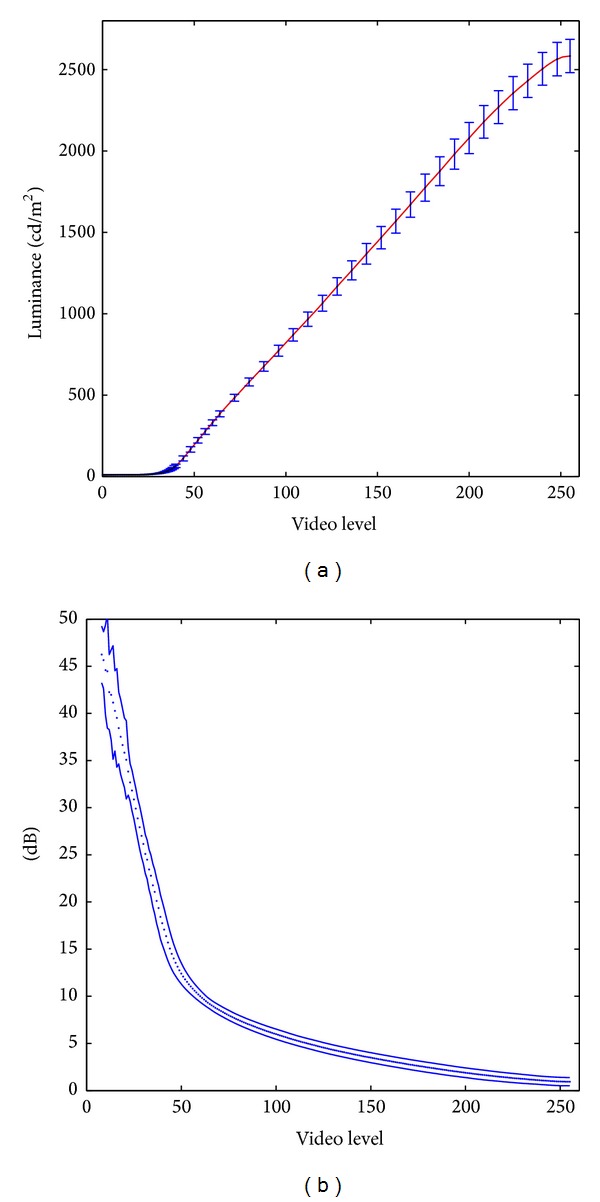
Example of luminance and dB calibration for OLED microdisplay. (a) Measured luminance versus video control level (gray level). Average luminance obtained in a series of 6 measurements performed over a period of 8 months with error bars representing the standard deviation. Solid red line is a cubic spline fit to the measured averaged data. (b) Stimulus intensity on the Humphrey decibel scale (dB) as a function of video level. Points represent dB values corresponding to consecutive integer video levels on 8-bit scale. Solid lines represent standard deviation associated with reproducibility of luminance measurements.

Luminance of the displays was measured (at the output of display optic) as a function of the image brightness given in bits, with 0 corresponding to black and 255 corresponding to white—Figure 3(a). The corresponding decibel (dB) levels are calculated in the same way as for the HFA reference instrument:

(1) $$\begin{array}{c} \text{dB}=10\;\log_{10}\left(\frac{L_{\max}}{L_{T}-L_{B}}\right), \end{array}$$

where *L*_max⁡_ is maximum stimulus luminance (for the reference HFA instrument, *L*_max⁡_ = 3183 cd/m^2^), *L*_*T*_ is the stimulus luminance, and *L*_*B*_ is the background luminance. Similar to HFA, *L*_*B*_ = 10 cd/m^2^ for the VirtualEye. The following observations are made.

1. Luminous output is not a linear function of the video level (gray level), especially for the low luminance levels.
2. On the HFA decibel scale, the stimulus range achievable with OLED displays is from 1–5 dB to about 45 dB (as compared to HFA's 0 to 50 dB), depending on the type (white or green monochrome, or color) and individual display. This is sufficient for glaucoma applications.  Figure 3(b) shows the dB range for one of the displays in the current prototype (green monochrome display).
3. High decibel (low luminance) limit is achieved by adjusting the display driver to produce a very slow increase in luminance at low video levels.
4. Low dB (high luminance) range is limited by maximum luminance of the display. In VirtualEye this is partially offset by an increase of stimulus size. The standard Goldman III stimulus angular size of 0.43 degrees is used up to the maximum luminance; then the size is slightly increased to achieve dB values down to zero.
5. Small variation in the output of the microdisplays was observed in repeat calibrations performed over a period of about 8 months—Figure 3(a). On the decibel (dB) scale, the maximum difference between six different calibrations was about 2 dB for intensities in 20–35 dB range and about 0.5 dB for intensities smaller than 10 dB. This uncertainty increases substantially (to about 4 dB) for intensities above 35 dB, but this region is of little importance from the diagnostic point of view.

### 3.2. Comparison with Standard Static Perimetry (HFA II)

Eighty-four participants were recruited and eighty took the VirtualEye test in manual mode and seventy-six in visual grasp mode. Seventy-nine patients successfully completed HFA II test: fifty-one took SITA Standard 24-2 test and twenty-eight took SITA Fast 30-2 test, from which 24-2 subset was extracted for further analysis. There were 59 eyes successfully tested with HFA II and VirtualEye in manual mode, 40 eyes tested with HFA II and VirtualEye in visual grasp mode, and 37 eyes successfully tested with VirtualEye in two modes—Table 1. The average test time was 10.6 ± 3.3 minutes for VirtualEye manual test and 9.4 ± 2.1 minutes for visual grasp test. This is somewhat longer than 6.1 ± 1.0 minutes for 51 SITA Standard tests performed for this study but comparable to usual duration of the full threshold test. (SITA Fast tests were performed on a larger 30-2 grid and thus the tests' times cannot be compared. However, SITA Fast is usually significantly shorter than SITA Standard.)

For VirtualEye, the following tests were considered not successful and were not used in analysis.

- Tests for 8 patients who were not able to complete the test in either mode due to difficulties in following the instructions, inability to properly place and adjust the visor on the head, or self-reported double vision and/or fatigue.
- Tests that were not completed due to technical problems with the prototype device including software malfunction (13 eyes tested in visual grasp mode), operator error leading to loss of data (4 patients and 1 patient in manual and visual grasp mode, resp.), and hardware failure during the test (1 patient).
- Tests with excessive number of fixation losses (>30%, correlated with excessive test time) and showing a large number (>30%) of false positives or false negatives (5 and 11 eyes for manual mode and visual grasp mode, resp.).
- Eyes with nonexisting or unreliable (>30% fixation losses, false positives or negatives) HFA II data (3 and 2 eyes, resp.).

As shown in Table 1, there was a large range of visual field defects in all groups (as measured by the median deviation, MD, determined with HFA II). Most of the subjects were diagnosed with glaucoma or suspect glaucoma, with some normal and neuroophthalmological diagnoses. The number of diagnoses in each group is shown in Table 2.

The retinal sensitivity measurements obtained with VirtualEye (VE) device were compared with those produced by HFA II. For each tested eye, we produced point-by-point comparisons between the available results: VE visual grasp (VG) versus HFA II, VE manual (MAN) versus HFA II, and VE visual grasp versus VE manual. Thus, for every pair-wise comparison, 52 values are produced representing the differences (in dB) between the two measurement fields, with the blind spot excluded.

**Figure 4:**
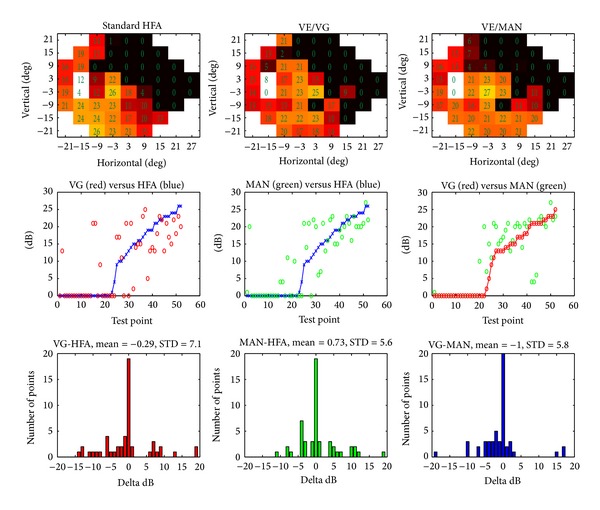
Case example of glaucoma with comparable retinal sensitivity values measured by standard (HFA) perimetry and VirtualEye in visual grasp (VE/VG) and manual (VE/MAN) modes. “Delta dB” denotes the difference in measured sensitivity between respective devices.

**Figure 5:**
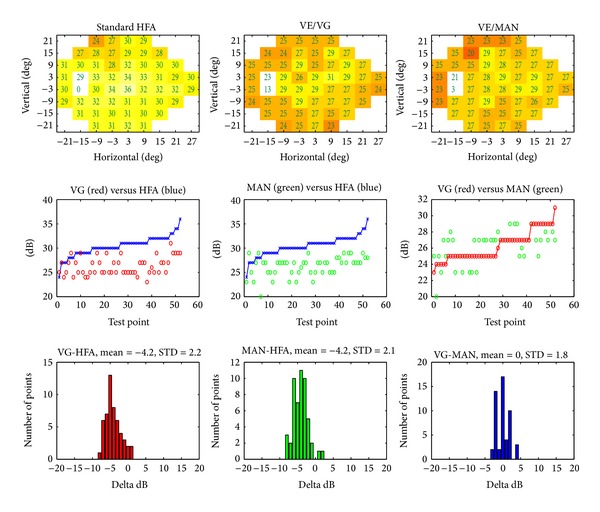
Example of results obtained for a normal eye, otherwise the same as Figure 4.

Figure 4 presents an example of data obtained for a glaucomatous eye and results of interplatform comparisons. Upper row panels show the measured sensitivities (color-coded, with dB values superimposed). The middle row graphs show values measured by the reference instrument (HFA, HFA, and VE/MAN, resp.), sorted by their ascending values, and values for the corresponding points measured by one other modality (VE/VG, VE/MAN, and VE/VG, resp.). The lower row graphs are histograms of point differences between the respective modalities. In this case, for about 20 measurement locations the difference between the two modalities depicted on each histogram is zero. The mean difference between the platforms is low (of the order of 1 dB), with standard deviation of differences between 7.1 dB (VG-MAN) and 5.6 (MAN-HFA).  Figure 5 is an example of data obtained for a normal eye, presented in the same fashion. In this case, there is a small systematic shift (about 4 dB) between the measurements obtained in both VE modes and HFA and good agreement between VE/VG and VE/MAN measurements.

**Figure 6:**
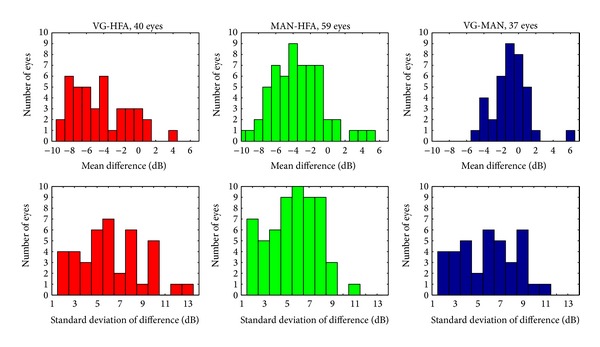
Summary of interplatform comparisons.

A summary of interplatform comparisons for all eyes, for which results from at least two modalities were available, is given in Figure 6. We show histograms of “Mean” and “STD” parameters (mean of the difference and its standard deviation) that are exemplified in lower row histograms in Figures 4 and 5. There appears to be a systematic shift between the mean sensitivity measurements obtained with VE and HFA modalities. The (average) standard deviation for difference in sensitivity measurements is of the order of 5 dB for all interplatform comparisons.

**Figure 7:**
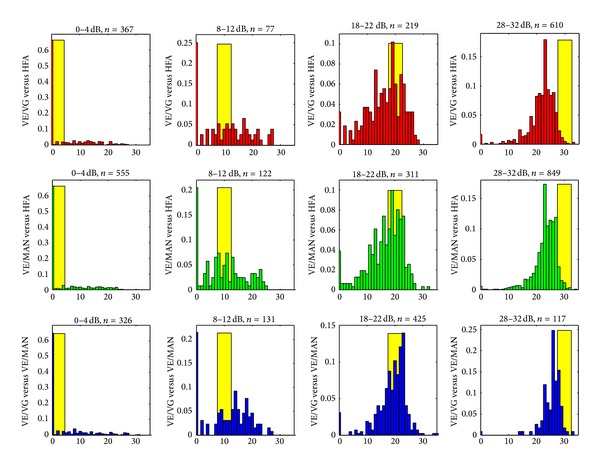
Distributions of retinal sensitivity measurements related to the reference platform test values in selected ranges (0–4 dB, 8–12 dB, 18–22 dB, and 28–32, yellow bars). Vertical axes: the fraction of measurements obtained in each 1 dB interval, horizontal axes: measured sensitivity (dB). “*n*” is the number of data points available for each comparison.

Figure 7 shows results of interplatform comparisons as distributions of measured sensitivities stratified by the reference platform measurements [15]. Here, the reference platforms are HFA (top two rows) and VE/MAN (the bottom row). The distributions of values produced by the tested modalities are presented as histograms with *y*-value equal to the fraction of measurements obtained in each 1 dB interval. The systematic shift in measured sensitivities between VE and HFA is most pronounced in the upper range of 28 to 32 dB.

### 3.3. Usability Testing

Seventy subjects completed the usability questionnaire. The questionnaire required graded responses on the scale from 1 to 5—Table 3. The responses summarized in Table 3 show a preference by the study participants for the head-mounted perimeter over the standard HFA II, due to improved comfort and ease of use. There was no clear preference for the VirtualEye test mode (manual versus visual grasp). The responses were not statistically different between groups of patients who successfully completed all visual field tests and those who did not or who completed only one of the VirtualEye tests.

## 4. Discussion

Preliminary results of clinical testing of the novel VirtualEye head-mounted perimeter indicate the ability to test the visual field in both standard manual and new visual grasp modes in a manner comparable to standard automated perimetry with the HVF analyzer.

The results of interplatform comparisons should be considered in the context of reproducibility of standard perimetric measurements. We note that measurement distributions plotted in Figure 7 are very similar to those obtained for HFA II (SITA) retest comparisons [15] with the only major difference being a systematic shift (about −5 dB) of VirtualEye sensitivities with respect to HFA II sensitivities. Thus, it appears that the majority of standard deviations between VirtualEye and HFA II results (as well as between two modes of the VirtualEye) may be attributed to the intrinsic reproducibility of perimetric measurements. The origin of systematic (average) shift towards lower sensitivities is not clear. It may be attributable to the differences in display technology between the standard and head-mounted devices. However, we also note that the shift was not observed for all patients, which may indicate differences in individual perception.

Different test strategies (SITA, SITA Fast used with HFA II, and full threshold (FT) used in VirtualEye) may produce somewhat different results for estimates of retinal sensitivity. For example, in a study by Artes et al. [16], the differences were found to be close to zero at both ends of the dynamic range and reached maximum at sensitivity of about 15 dB, where the difference between SITA and FT was found to be about 1.5 dB and 2.5 dB for the SITA Standard and Fast strategies, respectively. However, these systematic differences are still smaller than test-to-test reproducibility of all considered strategies. Thus, the use of different test strategies is expected to have only a small effect on the interinstrument comparisons presented here.

There are several limitations of the current research. The VirtualEye tests in visual grasp mode often indicated poor fixation and/or excessive number of false positives or false negatives (15% of eyes). This may be due to the different mode of operation, requiring relearning of responses for patients already accustomed to the standard automatic perimetry. All subjects were experienced with perimetry but only in standard manual mode with HFA testing. Although brief training was performed for the visual grasp mode, it may be argued that more familiarity with that mode could result in better performance. Also, as this test was usually performed at the end of the perimetry session, patient fatigue may have played a role. The tests were not randomly ordered, and this may result in superior performance for earlier testing, favoring standard automated perimetry, then manual mode with the study device, and finally with visual grasp mode with the study device. A relatively large number of software failures (17% of cases) in the visual grasp mode were seen and led to improvements in the development of the test algorithm. Larger trials may be considered with perimetry naïve participants, with random order of testing.

The patient questionnaire is helpful in gauging the initial acceptance of the virtual reality testing concept and visual grasp mode. However, participants in a trial may be more willing to try novel testing and also feel obligated to give a more positive response.

The eye tracker permits strict monitoring of the subject gaze direction (fixation), which is potentially more accurate than standard perimetry where fixation is checked only occasionally during the test (typically, using the blind spot location as a control point). In the current implementation of the testing software for the manual mode (where tracker is used only for fixation check), the threshold for fixation loss is conservatively set at about 3 degrees (about half of the angular distance between two adjacent test points). It is not clear if continuous control of the fixation direction (checked before each stimulus) has some influence on the overall accuracy of the visual field determination. Although HFA II also has the gaze-tracking capability, different criteria for fixation loss may be another source of discrepancies between HFA II and VirtualEye measurements, at least for some patients. It appears that continuous control of fixation direction available in VirtualEye should be especially useful in high-resolution perimetry [17].

## 5. Conclusions

The present research is a proof of concept study exploring the practicality, reliability, and validity of head-mounted, virtual reality perimetry measurements. The initial clinical results indicate agreement of visual field measurements taken by the VisualEye head-mounted perimeter in both manual and visual grasp modes and the HFA test. The observed differences are mostly consistent with previously observed reproducibility of the perimetric examination. The study appears to validate both of the concepts of a head-mounted, virtual reality type perimeter as well as the visual grasp mode using eye tracking instead of manual patient response. This new modality of automated perimetry may prove useful in a wide range of patients, including those who have compromised access to, or limited abilities in, using the standard testing paradigm.

## Figures and Tables

**Table 1:** Age and visual field defect severity for three groups of data used for interplatform comparisons.

	Number of eyes	Age				MD			
		Mean	STD	Median	Range	Mean	STD	Median	Range
VG versus HFA	40	63	13	61	37–82	−8.8	9.1	−6.0	−32.5–1.4
MAN versus HFA	59	63	13	66	35–82	−9.2	8.8	−7.4	−32.5–1.4
VG versus MAN	37	62	13	61	37–82	−8.6	9.0	−6.1	−32.5–1.4

STD—standard deviation. MD—mean deviation (as determined by HFA II test).

**Table 2:** Ophthalmic diagnoses for the study subjects.

Diagnosis	Number of subjects
Primary open angle glaucoma	22
Low tension glaucoma	3
Narrow angle glaucoma	3
Chronic angle closure glaucoma	1
Pigmentary glaucoma	1
Glaucoma suspect	6
Pseudophakia	1
Optic nerve drusen	2
Autosomal dominant optic atrophy	1
Cortical stroke and tumor	2
Multiple sclerosis/optic neuritis	2
Craniopharyngioma	1
Normal	17

**Table 3:** Summary of usability questionnaire responses.

Usability question	Median rating and % of responses for each rating (1 through 5)
Overall comfort (1—very comfortable, 5—very uncomfortable)	1 $\begin{array}{ccccc} {}[51 & 21 & 17 & 9 & 2] \end{array}$
Comfort in comparison with HFA (1—much better, 5—much worse)	2 $\begin{array}{ccccc} {}[39 & 29 & 18 & 10 & 4] \end{array}$
Were the test principles easy to understand? (1—easy, 5—difficult)	1 $\begin{array}{ccccc} {}[76 & 11 & 6 & 6 & 1] \end{array}$
Overall difficulty of test procedure (1—very easy, 5—very difficult)	1 $\begin{array}{ccccc} {}[56 & 28 & 10 & 4 & 2] \end{array}$
Device preference (1—VirtualEye, 5—HFA)	2 $\begin{array}{ccccc} {}[48 & 16 & 14 & 9 & 13] \end{array}$
VirtualEye mode preference (1—manual, 5—visual grasp)	3 $\begin{array}{ccccc} {}[40 & 7 & 14 & 5 & 34] \end{array}$
